# Housing affordability and psychological distress: Household measures mirror individual estimates but miss one in three at-risk adults

**DOI:** 10.1016/j.pmedr.2026.103556

**Published:** 2026-07-02

**Authors:** Kazuya Ogawa, Keiichi Shimatani, Kohki Takaguchi, Norimichi Suzuki

**Affiliations:** a Center for Preventive Medical Sciences, Chiba University, 1-33 Yayoi-Cho, Inage-Ku, Chiba 263-8522, Japan; b Design Research Institute, Chiba University, 1-19-1, Bunka, Sumida-ku, Tokyo 131-0044, Japan

**Keywords:** Mental health, Housing costs, Housing tenure, Mortgage, Japan

## Abstract

**Objective:**

Conventional household-based housing affordability measures assume equal burden-sharing, potentially diluting or obscuring health risks of individuals with disproportionate burdens. To examine the implications of this assumption in research and preventive policy, we investigated the respective associations of household and individual housing affordability with psychological distress.

**Methods:**

Analyzing data from a web-based cross-sectional survey conducted in November 2025 (6619 Japanese adults), we assessed classification discordance between household and individual housing unaffordability (cost-to-income ratio ≥ 30%) and examined their associations with psychological distress (Kessler Psychological Distress Scale ≥5). Modified log Poisson regression estimated prevalence ratios by housing tenure, using multiple imputation.

**Results:**

Adjusted prevalence ratios (aPR) were similar for the household (1.15; 95% confidence interval [CI]: 1.05, 1.26) and individual (1.17; 95% CI: 1.06, 1.28) indicators. Stratification by housing tenure showed strong associations for both indicators among homeowners (both aPR = 1.33), whereas no significant associations were observed among renters. However, the household measure overlooked about a third of individuals with unaffordable housing.

**Conclusions:**

While the assumption of equal burden-sharing appears to be sufficient for research, it may obscure specific vulnerabilities when used for screening. Preventive strategies could be refined by incorporating individual indicators to capture these hidden risks.

## Highlights

• Household and individual housing affordability showed comparable links to distress.
• These associations were observed among owners but not renters.
• Household indicators may serve as practical alternatives for research.
• Yet, household measures missed 30% of individuals with unaffordable housing.
• Individual metrics may complement household ones by capturing hidden risks.

## 1 Introduction

Housing serves as a fundamental social determinant of health, standing as a critical pillar alongside housing security and housing suitability (Bentley et al., 2025). Within this framework, empirical research has extensively examined the association between housing affordability and mental health. Specifically, unaffordable housing has been associated with depressive symptoms (Kim et al., 2021; Park and Jung, 2019; Park and Seo, 2023; Park and Seo, 2022; Wang et al., 2023), psychological distress (Ogawa et al., 2025), and general mental health indicators (Arundel et al., 2024; Baker et al., 2014; Bentley et al., 2022; Bentley et al., 2011; Mason et al., 2013).

Previous literature has primarily employed two definitions of housing affordability. The first definition utilizes the household housing cost-to-income ratio with a 30% cutoff (Bentley et al., 2016b; Bentley et al., 2011; Jurčišinová et al., 2025; Kavanagh et al., 2016; Kim et al., 2021; Mason et al., 2013; Ogawa et al., 2026; Ogawa et al., 2025; Park and Jung, 2019; Park and Seo, 2023; Park and Seo, 2022; Wang et al., 2023). The second, known as the “30/40 rule,” applies this 30% criterion exclusively to households falling within the lowest 40% of the income distribution (Arundel et al., 2024; Baker et al., 2014; Bentley et al., 2022; Bentley et al., 2019; Botha et al., 2024; Singh et al., 2020).

However, these measurement approaches present methodological limitations regarding their alignment with some housing systems. While existing definitions typically utilize household-level measurements, which assume equal burden-sharing among household members, financial assessment for housing in Japan primarily depends on individual income. Specifically, mortgages assessed using individual income (solo and dual) account for 87% of the total, whereas those based on aggregation account for 13% (Japan Housing Finance Agency, 2025). Consequently, conventional definitions relying solely on household-level indicators may not fully capture the individual economic realities determining housing access.

Furthermore, the concentration of housing costs on specific individuals poses challenges for both policy and research. From a preventive policy perspective, household-level metrics risk masking individual heterogeneity, as the household aggregate may be classified as affordable even if an individual bears an excessive burden. Consequently, these definitions may undermine targeted prevention efforts, leaving individuals with hidden burdens unprotected.

From a methodological perspective, concerns persist regarding whether household housing affordability captures individual mental health risks. Household-level indicators may dilute and underestimate the risks faced by high-burden individuals because of low-burden household members (Bentley et al., 2016a, Bentley et al., 2016b). Indeed, research utilizing individual-level burden has reported associations predominantly among men (Ogawa et al., 2026), suggesting that gender norms (e.g., the male breadwinner model) may generate non-uniform burdens and health implications within the same household. Although household metrics could serve as a useful alternative measure if financial stress is shared equally (Gathergood, 2012), the predominant reliance on these metrics means that it remains unclear whether they closely approximate individual burden as assumed, or systematically underestimate individual risk.

Despite these concerns regarding the assumption of equal burden-sharing, empirical assessments in previous research have been largely limited to household-level measures (e.g., Bentley et al., 2011; Jurčišinová et al., 2025). Therefore, we aimed to examine the respective associations of household and individual housing affordability with psychological distress among homeowners and renters.

## 2 Methods

### 2.1 Study design and population

This web-based cross-sectional study utilized a survey platform provided by Rakuten Insight, Inc., which maintains a panel of approximately 2.25 million registered potential respondents. The survey, conducted from November 10 to 13, 2025, targeted individuals aged 18 years or older. Quota sampling based on 2021 Population Estimates (age, gender, and prefecture) was performed to align the sample distribution with the Japanese demographic structure (Ministry of Internal Affairs and Communications, 2021), resulting in the distribution of survey requests to 208,473 randomly selected monitors, and yielded a dataset of 7000 participants. Survey completers received points as an incentive. In the data-cleaning step, we excluded “speeders” whose response times were below half the median time (Leiner, 2019). These procedures yielded a final analytic sample of 6619 participants.

### 2.2 Measures

#### 2.2.1 Outcome variable

The Kessler Psychological Distress Scale (K6) was used to assess psychological distress (Kessler et al., 2002). The K6 measures the frequency of six emotional states (nervous, hopeless, restless or fidgety, depressed, everything was an effort, worthless) over the past 30 days. Items are rated on a 5-point scale (0–4), with higher total scores (range: 0–24) indicating more severe distress. Based on Japanese validation studies recommending a clinical screening threshold (Sakurai et al., 2011), the total score was dichotomized into “psychological distress” (≥ 5 points) and “no distress” (< 5 points).

#### 2.2.2 Exposure variables

Primary exposure variables consisted of two housing affordability indicators: (1) household housing affordability, defined as the ratio of monthly household housing costs to monthly household income, and (2) individual housing affordability, defined as the ratio of monthly individual housing costs to monthly individual income. Housing costs were assessed through a four-step sequence. First, respondents identified whether their household's primary housing costs consisted of rent (including employer-provided housing) or mortgage payments; those whose households paid neither did not proceed to the subsequent housing cost questions and were assigned zero housing costs. Second, respondents reported the total monthly amount paid by their household as rent or mortgage payments in a typical month (excluding bonus months). Third, respondents indicated whether they personally bore any of these costs. Fourth, respondents who bore any of these costs reported the amount they personally paid each month. Both household and individual housing costs were collected as continuous variables through direct numerical entry. Housing costs in this study were limited to rent or mortgage payments and did not include property taxes or utilities. Individual and household income were each assessed separately as gross annual income, including bonuses and supplementary income. Annual income, collected in 28 categories (e.g., point estimates such as approximately 5 million Japanese yen), was converted to monthly values using the representative values. Following previous literature (Bentley et al., 2011; Mason et al., 2013; Ogawa et al., 2026; Park and Seo, 2023), each indicator was dichotomized into “Unaffordable” (housing cost-to-income ratio ≥ 30%) and “Affordable” (< 30%).

#### 2.2.3 Control variables

The models incorporated the following covariates as potential confounders. Demographic variables included age (continuous), gender (female, male), and marital status (married, unmarried, widowed or divorced). Socioeconomic status variables comprised educational attainment (less than university, university or higher), employment status (employed, unemployed), and household annual income (quartiles). Additionally, household and environmental variables included household size (single-person, multi-person) and population size of the area of residence (small, middle, large, metropolitan area).

### 2.3 Statistical analysis

#### 2.3.1 Multiple imputation

With missing data rates ranging from 0% to 21% across variables, the analysis employed multiple imputation. The imputation models included all variables used in the analysis. Variable specification treated age and housing costs as continuous; K6 scores, education, income, household size, and population size were treated as ordinal; and other variables were treated as nominal. The Amelia statistical package (Honaker et al., 2011) generated 30 imputed datasets, with results pooled according to Rubin's rules. Convergence diagnostics and comparison of distributions between observed and imputed data were used to assess model fit.

#### 2.3.2 Main analysis

Descriptive statistics utilized the original pre-imputation data, summarizing participant characteristics by tenure type.

The main analysis proceeded in two stages using the pooled imputed data. First, the analysis assessed the extent to which the household measure captures individuals with unaffordable housing. Second, these indicators were entered into separate models stratified by housing tenure (homeowners vs. renters) to estimate unadjusted and adjusted associations. Given the high outcome prevalence (> 10%), odds ratios from logistic regression may overestimate the prevalence ratio (PR). Consequently, the analysis utilized modified Poisson regression with robust error variance to directly estimate PRs (Gallis and Turner, 2019; Zou, 2004). To evaluate the robustness of the main findings, sensitivity analyses repeated the procedure using alternative housing affordability cutoffs of 20%, 25%, 35% and 40%. All analyses were conducted using R version 4.3.3 (Amelia package version 1.8.3).

### 2.4 Ethical statement

This study received approval from The Research Ethics Committee of the Graduate School of Medicine, Chiba University (Approval No. M10850) and adhered to the principles of the Declaration of Helsinki. All participants provided web-based informed consent prior to the survey.

## 3 Results

### 3.1 Descriptive statistics

Table 1 shows the sociodemographic characteristics of the 6619 study participants. Homeowners comprised 67.9% of the sample, while renters accounted for 32.1%. A demographic comparison showed that homeowners exhibited a higher mean age (55.6 vs. 44.2 years) and a higher proportion of married individuals (68.0% vs. 39.6%). Conversely, renters exhibited higher levels of educational attainment (university degree or higher: 52.0% vs. 47.6%), a higher employment rate (75.2% vs. 60.2%), and a greater prevalence of single-person households (46.4% vs. 11.4%).

**Table 1:** Characteristics of homeowners and renters in a web-based survey in Japan, 2025, stratified by housing tenure.

Characteristics	Overall	Homeowner	Renter	*p*-value
	*N* = 6619	*N* = 4496	*N* = 2123	
Psychological distress				< 0.01
No	3755 (56.7)	2761 (61.4)	994 (46.8)	
Yes	2864 (43.3)	1735 (38.6)	1129 (53.2)	
Missing	0	0	0	
Household housing affordability				< 0.01
Affordable (< 30%)	4796 (91.5)	3364 (96.2)	1432 (82.2)	
Unaffordable (≥ 30%)	444 (8.5)	133 (3.8)	311 (17.8)	
Missing	1379	999	380	
Individual housing affordability				< 0.01
Affordable (< 30%)	5385 (93.3)	3772 (96.5)	1613 (86.5)	
Unaffordable (≥ 30%)	388 (6.7)	136 (3.5)	252 (13.5)	
Missing	846	588	258	
Age, mean (SD)	51.9 (16.0)	55.6 (15.3)	44.2 (14.8)	< 0.01
Missing	0	0	0	
Gender				0.80
Female	3306 (50.3)	2243 (50.2)	1063 (50.6)	
Male	3262 (49.7)	2225 (49.8)	1037 (49.4)	
Missing	51	28	23	
Marital status				< 0.01
Married	3866 (59.0)	3041 (68.0)	825 (39.6)	
Unmarried	1972 (30.1)	985 (22.0)	987 (47.4)	
Widowed or divorced	716 (10.9)	445 (10.0)	271 (13.0)	
Missing	65	25	40	
Educational attainment				< 0.01
Less than university	3344 (51.0)	2341 (52.4)	1003 (48.0)	
University or higher	3208 (49.0)	2123 (47.6)	1085 (52.0)	
Missing	67	32	35	
Employment status				< 0.01
Employed	4304 (65.0)	2707 (60.2)	1597 (75.2)	
Unemployed	2315 (35.0)	1789 (39.8)	526 (24.8)	
Missing	0	0	0	
Household size				< 0.01
Single-person	1497 (22.6)	511 (11.4)	986 (46.4)	
Multi-person	5122 (77.4)	3985 (88.6)	1137 (53.6)	
Missing	0	0	0	
Household income				< 0.01
Low	1545 (29.5)	956 (27.3)	589 (33.8)	
Mid-low	1257 (24.0)	791 (22.6)	466 (26.7)	
Mid-high	1284 (24.5)	859 (24.6)	425 (24.4)	
High	1154 (22.0)	891 (25.5)	263 (15.1)	
Missing	1379	999	380	
Individual income				< 0.01
Low	1721 (29.8)	1260 (32.2)	461 (24.7)	
Mid-low	1934 (33.5)	1314 (33.6)	620 (33.2)	
Mid-high	904 (15.7)	524 (13.4)	380 (20.4)	
High	1214 (21.0)	810 (20.7)	404 (21.7)	
Missing	846	588	258	
Population size				< 0.01
Small	723 (10.9)	526 (11.7)	197 (9.3)	
Middle	3155 (47.7)	2314 (51.5)	841 (39.6)	
Large	1383 (20.9)	883 (19.6)	500 (23.6)	
Metropolitan	1358 (20.5)	773 (17.2)	585 (27.6)	
Missing	0	0	0	

Abbreviations: SD, standard deviation.

Values are numbers (percentages) unless otherwise indicated.

*P*-values were calculated using Pearson's Chi-squared test for categorical variables and the Kruskal–Wallis test for continuous variables.

Regarding economic indicators, distinct patterns emerged: whereas homeowners reported higher household income than renters (highest quartile: 25.5% vs. 15.1%), individual income levels remained comparable between the two groups (highest quartile: 20.7% vs. 21.7%). The overall prevalence of psychological distress was 43.3%; stratification by tenure revealed a higher prevalence among renters (53.2%) compared with homeowners (38.6%). Similarly, renters exhibited higher rates of unaffordable housing across both the household indicator (17.8% vs. 3.8%) and the individual indicator (13.5% vs. 3.5%).

### 3.2 Housing affordability and psychological distress

In the first stage of the main analysis, we evaluated the extent to which the household measure captures individuals with unaffordable housing in the pooled imputed data (Table 2). Of the 464.7 individuals classified as having unaffordable housing by the individual measure, 146.3 (31.5%) were classified as affordable by the household measure.

**Table 2:** Agreement between individual and household housing affordability measures among adults: a web-based survey in Japan, 2025.

	Individual housing affordability	
Household housing affordability	<30%	≥30%
<30%	5898.0 (95.8%)	146.3 (31.5%)
≥30%	256.3 (4.2%)	318.4 (68.5%)
Total	6154.3 (100.0%)	464.7 (100.0%)

Values represent the pooled average counts and column percentages across the multiply imputed datasets.

Table 3 shows the associations between housing affordability and psychological distress. In the overall sample, unaffordable housing was associated with a higher prevalence of psychological distress across both household and individual indicators. Although adjustment for confounders attenuated the magnitude of association, statistical significance persisted. The adjusted prevalence ratios (aPR) were 1.15 (95% confidence interval [CI]: 1.05, 1.26) for the household indicator and 1.17 (95% CI: 1.06, 1.28) for the individual indicator.

**Table 3:** Prevalence ratios of psychological distress among adults by housing tenure and housing affordability using a 30% cut-off for housing costs relative to income: a web-based survey in Japan, 2025.

	Household housing affordability		Individual housing affordability	
	Unadjusted	Adjusted	Unadjusted	Adjusted
	PR [95% CI]	PR [95% CI]	PR [95% CI]	PR [95% CI]

Overall				
Affordable (< 30%)	1.00	1.00	1.00	1.00
Unaffordable (≥ 30%)	1.41 [1.30, 1.52]	1.15 [1.05, 1.26]	1.31 [1.19, 1.43]	1.17 [1.06, 1.28]

Homeowner				
Affordable (< 30%)	1.00	1.00	1.00	1.00
Unaffordable (≥ 30%)	1.71 [1.51, 1.93]	1.33 [1.16, 1.51]	1.53 [1.33, 1.76]	1.33 [1.14, 1.54]

Renter				
Affordable (< 30%)	1.00	1.00	1.00	1.00
Unaffordable (≥ 30%)	1.07 [0.97, 1.19]	1.00 [0.89, 1.13]	1.02 [0.90, 1.14]	1.01 [0.89, 1.14]

Abbreviations: PR, prevalence ratio; CI, confidence interval.

Data from modified log Poisson regression with affordable housing (< 30% of income) as reference.

Adjusted models controlled for age, gender, marital status, educational attainment, employment status, household size, annual household income, population size.

Estimates were pooled from 30 imputed datasets using Rubin's rules.

Stratified analysis, however, showed distinct patterns contingent on tenure type. Homeowners consistently exhibited stronger associations than the overall sample. In the adjusted models, the magnitude of association remained comparable for both the household indicator (aPR 1.33; 95% CI: 1.16, 1.51) and the individual indicator (aPR 1.33; 95% CI: 1.14, 1.54). In contrast, renters showed no significant association in the models for either indicator. The aPRs were close to the null value, with CIs including unity (Household aPR 1.00, 95% CI: 0.89, 1.13; Individual aPR 1.01, 95% CI: 0.89, 1.14).

Table 4 summarizes the sensitivity analyses using alternative cutoffs of 20%, 25%, 35%, and 40%. The aPRs in the overall sample ranged from 1.10 to 1.15 for the household indicator and from 1.12 to 1.17 for the individual indicator across the four thresholds, with the tenure-specific patterns consistent across all thresholds.

**Table 4:** Prevalence ratios of psychological distress among adults by housing tenure and housing affordability using 20%, 25%, 35%, and 40% cut-offs for housing costs relative to income: a web-based survey in Japan, 2025.

	20%		25%		35%		40%	
	Household	Individual	Household	Individual	Household	Individual	Household	Individual
	Adjusted	Adjusted	Adjusted	Adjusted	Adjusted	Adjusted	Adjusted	Adjusted
	PR [95% CI]	PR [95% CI]	PR [95% CI]	PR [95% CI]	PR [95% CI]	PR [95% CI]	PR [95% CI]	PR [95% CI]

Overall								
Affordable	1.00	1.00	1.00	1.00	1.00	1.00	1.00	1.00
Unaffordable	1.10 [1.02, 1.19]	1.12 [1.03, 1.20]	1.16 [1.06, 1.26]	1.15 [1.06, 1.26]	1.13 [1.03, 1.25]	1.16 [1.04, 1.28]	1.11 [1.00, 1.24]	1.17 [1.04, 1.30]

Homeowner								
Affordable	1.00	1.00	1.00	1.00	1.00	1.00	1.00	1.00
Unaffordable	1.19 [1.06, 1.33]	1.20 [1.06, 1.35]	1.25 [1.10, 1.42]	1.26 [1.10, 1.45]	1.33 [1.14, 1.55]	1.37 [1.16, 1.61]	1.34 [1.14, 1.57]	1.35 [1.14, 1.61]

Renter								
Affordable	1.00	1.00	1.00	1.00	1.00	1.00	1.00	1.00
Unaffordable	0.99 [0.90, 1.10]	1.01 [0.92, 1.12]	1.06 [0.95, 1.18]	1.03 [0.92, 1.15]	1.00 [0.89, 1.13]	0.99 [0.86, 1.13]	0.98 [0.86, 1.12]	1.00 [0.86, 1.16]

Abbreviations: PR, prevalence ratio; CI, confidence interval.

Data from modified log Poisson regression with affordable housing (below each cut-off) as reference.

Adjusted models controlled for age, gender, marital status, educational attainment, employment status, household size, annual household income, population size.

Estimates were pooled from 30 imputed datasets using Rubin's rules.

## 4 Discussion

The respective associations of household and individual housing affordability with psychological distress were of comparable magnitudes. Furthermore, the strong association observed among homeowners in both models suggests that mortgage burdens may represent a critical social determinant of health. Despite these similar estimates, the household measure misclassified about one in three at-risk individuals as affordable. This misclassification suggests that while conventional household-level indicators can serve as a practical alternative for population-level estimates, they obscure specific individual risks.

We observed consistency between estimates from household and individual indicators. While previous research has raised concerns that household-level aggregation might dilute individual financial burden and underestimate health risks (Bentley et al., 2016a, Bentley et al., 2016b), our data showed estimates of comparable magnitude for both measures. Specifically, aPRs were closely aligned in both the overall analysis (Household aPR 1.15; Individual aPR 1.17) and the homeowner stratum (1.33 for both). As a supplementary analysis addressing economies of scale, the household affordability measure was recalculated using equivalized household income. The overall aPR remained comparable (aPR 1.13, 95% CI: 1.05, 1.21), while the estimate among homeowners was slightly attenuated (aPR 1.19, 95% CI: 1.08, 1.32). Despite this attenuation, the overall pattern remained consistent (Supplementary Table 1). These findings suggest that conventional household indicators may provide reasonable approximations of individual housing cost burdens, addressing the concerns of underestimation raised in prior studies.

The associations observed in this study remained consistent with previous literature, even compared with studies utilizing varying outcome measures such as the Mental Health Inventory or Center for Epidemiologic Studies Depression Scale (Ogawa et al., 2026; Park and Seo, 2023). The magnitude of the association (aPR = 1.33) also generally aligned with the results of prior research, reaching levels comparable to or higher than those reported for high-income homeowners in Japan (aPR = 1.23) (Ogawa et al., 2025). However, the association specific to homeowners contrasts with some international findings. While our results are in accord with reports from the United Kingdom (Bentley et al., 2016b), China (Wang et al., 2023), and recent studies of mortgage burden in Japan (Ogawa et al., 2025) and Finland (Damiens et al., 2026), they differ from the findings of studies from the Netherlands (Arundel et al., 2024), Australia (Mason et al., 2013), and South Korea (Park and Jung, 2019), which have reported associations primarily among renters.

Institutional variations in rental protection systems across countries may explain the inconsistency between the current findings and previous reports. Previous research suggests that institutional protections, such as housing allowances, influence the mental health of renters (Bentley et al., 2016b). Despite the near absence of a public housing allowance system in Japan's private rental sector (Forrest and Hirayama, 2015; Hirayama, 2021), tenants are afforded strong legal protections under the Act on Land and Building Leases (Act No. 90 of 1991), which restricts lease termination without just cause and limits rent increases. This legal security of tenure may mitigate the psychological strain associated with housing cost burden among renters, partially explaining the null association observed in the present study. An alternative explanation involves selective residential mobility. Rental contracts, unlike mortgage obligations, offer flexibility through relocation. Consequently, renters reaching financial or psychological limits due to high housing costs may have already moved to cheaper housing or parental homes by the time of the survey, effectively exiting the unaffordable category (≥ 30%) and entering the affordable group (< 30%). This selective attrition would leave the unaffordable group composed predominantly of renters who can tolerate high housing costs without psychological deterioration, attenuating the association toward the null. Relocation to more affordable housing may also facilitate mental health recovery among movers, further reducing the contrast between groups.

Among homeowners, by contrast, the association observed may reflect the financial strain inherent in Japan's mortgage system. Although the government implements support measures such as tax deductions, our analysis showed an association with psychological distress specifically among the homeowners who are the intended beneficiaries of these schemes. Prior evidence supports financial hardship as a key pathway. An Australian mediation analysis indicated that financial hardship accounted for more than half of the association between unaffordable housing and mental health (Singh et al., 2020). The structural characteristics of the Japanese mortgage system may contribute to financial hardship among homeowners. Unlike rent payments, mortgage obligations entail a long-term debt liability (Drentea and Reynolds, 2012). Japanese mortgages are further characterized by repayment periods averaging approximately 30 years (Ministry of Land, Infrastructure, Transport and Tourism, 2023) and the prevalence of full recourse loans, which retain the obligation to repay remaining debt even after the sale of the property. The strong legal and economic binding force generated by this system may create pressure that offsets or even surpasses the psychological protective effects of residential stability intended by homeownership policies. This interpretation aligns with government surveys indicating that approximately 65% of households with mortgages report a sense of financial burden (Ministry of Land, Infrastructure, Transport and Tourism, 2023). The persistence of the association specifically among homeowners even at the relatively low threshold of 25% in the sensitivity analysis reinforces the notion that the quality of long-term debt obligations, rather than merely the quantity of burden, is relevant to psychological distress.

The first three limitations concern internal validity and are likely to bias the estimates upward. First, the cross-sectional design precludes causal inference. Specifically, reverse causality—by which psychological distress leads to income loss and subsequent unaffordable housing—cannot be excluded. Second, unmeasured confounders, such as local housing market prices and asset status, may have confounded the results. Third, the reliance on between-person effects prevented the elimination of bias from time-invariant unobserved heterogeneity, such as individual personality or values, which typically yields larger estimates than within-person effects. Future research should therefore employ longitudinal data to examine within-person effects using fixed-effects models. Conversely, the fourth and fifth limitations may underestimate the associations. Fourth, housing costs were limited to rent or mortgage payments, excluding property taxes and utilities, potentially underestimating housing cost burden. Fifth, housing affordability may carry different implications for residual income depending on income level, though a stratified analysis was limited by the small number of higher-income participants with unaffordable housing. Sixth, the data lacked information on mortgage origination year, precluding a sensitivity analysis restricted to recent mortgage holders to reduce heterogeneity in the temporal gap between current income and income at origination. Seventh, the nature of web-based surveys may have attracted participants with higher socioeconomic status. Compared with national statistics, the sample showed higher rates of university education (48.9% vs. 21.3%) and homeownership (68.0% vs. 61.4%) (Ministry of Internal Affairs and Communications, 2020). The underrepresentation of vulnerable populations suggests that the actual risk might be conservative. In particular, the difference between household-level and individual-level housing affordability measures may be larger in the general population, as the individual-level association with psychological distress could be stronger in populations where household income does not buffer individual economic strain. Thus, whereas internal validity issues may inflate the estimates, the limited scope of housing cost measures and the exclusion of high-risk populations are likely to exert a countervailing downward bias, suggesting the need to consider these opposing forces.

The findings of this study have critical implications for both public health research and housing policy. Methodologically, this study provides evidence supporting the conventional reliance on household-level indicators. The respective associations of household and individual housing affordability with psychological distress showed similar patterns, suggesting that existing household indicators can function as a practical alternative for representing individual housing cost burden. This finding is aligned with previous research positing that financial shocks are shared within households (Gathergood, 2012). Therefore, the use of household indicators as practical alternatives appears to be reasonable even when assessing individual-level outcomes. However, studies focusing on gender roles or intra-household inequality should still prioritize individual indicators, because household metrics risk masking individual burdens (Ogawa et al., 2026).

From a preventive policy perspective, the findings suggest the potential value of augmenting assessment frameworks. Although household indicators are widely utilized in previous research (Mason et al., 2013; Park and Seo, 2023), their direct application to screening leaves a substantial number of at-risk individuals undetected: in our analysis, the household measure overlooked about one in three individuals with unaffordable housing. Using equivalized household income reduced this proportion to approximately one in six, though a non-negligible share remained undetected (Supplementary Table 2). Therefore, preventive strategies could be enhanced by incorporating individual-level indicators for screening, particularly in contexts where housing access depends on individual income.

## 5 Conclusions

The current study provides insight into the nuanced implications of using household-level indicators. In epidemiological research, household indicators can function as practical alternatives, yielding estimates that are comparable to those from individual indicators. In contrast, for preventive screening, household indicators, by their nature, may mask individual burdens, suggesting the value of introducing individual-level indicators to optimize targeted prevention.

## CRediT authorship contribution statement

**Kazuya Ogawa**: Conceptualization, Data curation, Methodology, Formal analysis, Writing – original draft, Writing - review & editing. **Keiichi Shimatani**: Investigation, Methodology, Writing - review & editing. **Kohki Takaguchi:** Investigation, Writing - review & editing. **Norimichi Suzuki**: Investigation, Writing - review & editing. All authors have read and approved the final version of the manuscript.

## Declaration of generative AI and AI-assisted technologies in the writing process

During the preparation of this work the author used Gemini in order to improve readability and enhance language clarity. After using this tool/service, the authors reviewed and edited the content as needed and take full responsibility for the content of the published article.

## Funding

This work was supported by the 10.13039/501100001691Japan Society for the Promotion of Science Grant-in-Aid for Research Activity Start-up (24K22989).

## Declaration of competing interest

The authors declare the following financial interests/personal relationships which may be considered as potential competing interests: Kazuya Ogawa and Keiichi Shimatani report that financial support was provided by Sekisui House, Ltd. The funder had no role in study design, data collection and analysis, decision to publish, or preparation of the manuscript. All other authors declare that they have no known competing financial interests or personal relationships that could have appeared to influence the work reported in this paper.

## Data Availability

The authors do not have permission to share data.

## References

[bb0005] Arundel R., Li A., Baker E., Bentley R. (2024). Housing unaffordability and mental health: dynamics across age and tenure. Int. J. Hous. Policy.

[bb0010] Baker E., Mason K., Bentley R., Mallett S. (2014). Exploring the bi-directional relationship between health and housing in Australia. Urban Policy Res..

[bb0015] Bentley R., Baker E., Mason K., Subramanian S.V., Kavanagh A.M. (2011). Association between housing affordability and mental health: a longitudinal analysis of a nationally representative household survey in Australia. Am. J. Epidemiol..

[bb0020] Bentley R., Baker E., LaMontagne A., King T., Mason K., Kavanagh A. (2016). Does employment security modify the effect of housing affordability on mental health?. SSM Popul. Health.

[bb0025] Bentley R., Pevalin D., Baker E., Mason K., Reeves A., Beer A. (2016). Housing affordability, tenure and mental health in Australia and the United Kingdom: a comparative panel analysis. Hous. Stud..

[bb0030] Bentley R., Baker E., Aitken Z. (2019). The “double precarity” of employment insecurity and unaffordable housing and its impact on mental health. Soc. Sci. Med..

[bb0035] Bentley R., Baker E., Ronald R., Reeves A., Smith S.J., Simons K., Mason K. (2022). Housing affordability and mental health: an analysis of generational change. Hous. Stud..

[bb0040] Bentley R., Mason K., Jacobs D., Blakely T., Howden-Chapman P., Li A., Adamkiewicz G., Reeves A. (2025). Housing as a social determinant of health: a contemporary framework. Lancet Public Health.

[bb0045] Botha F., Bentley R., Li A., Wiesel I. (2024). Housing affordability stress and mental health: the role of financial wellbeing. Aust. Econ. Pap..

[bb0050] Damiens J., Berger M., Junna L., Martikainen P. (2026). Mortgage affordability and mental healthcare use: evidence from Finnish population registers. Soc. Sci. Med..

[bb0055] Drentea P., Reynolds J.R. (2012). Neither a borrower nor a lender be: the relative importance of debt and SES for mental health among older adults. J. Aging Health.

[bb0060] Forrest R., Hirayama Y. (2015). The financialisation of the social project: embedded liberalism, neoliberalism and home ownership. Urban Stud..

[bb0065] Gallis J.A., Turner E.L. (2019). Relative measures of Association for Binary Outcomes: challenges and recommendations for the Global Health researcher. Ann. Glob. Health.

[bb0070] Gathergood J. (2012). Debt and depression: causal links and social norm effects. Econ. J..

[bb0075] Hirayama Y. (2021). Housing, family, and life-course in post-growth Japan. Jpn. Archit. Rev..

[bb0080] Honaker J., King G., Blackwell M. (2011). Amelia II: a program for missing data. J. Stat. Softw..

[bb0085] Japan Housing Finance Agency (2025). Annual report on the users survey (in Japanese). https://www.jhf.go.jp/about/research/loan/user/index.html.

[bb0090] Jurčišinová V., Forbes C.S., Ng J.W.J., Želinský T. (2025). The mediating role of financial well-being in the relationship between housing affordability and mental health. Sci. Rep..

[bb0095] Kavanagh A.M., Aitken Z., Baker E., LaMontagne A.D., Milner A., Bentley R. (2016). Housing tenure and affordability and mental health following disability acquisition in adulthood. Soc. Sci. Med..

[bb0100] Kessler R.C., Andrews G., Colpe L.J., Hiripi E., Mroczek D.K., Normand S.-L.T., Walters E.E., Zaslavsky A.M. (2002). Short screening scales to monitor population prevalences and trends in non-specific psychological distress. Psychol. Med..

[bb0105] Kim S.H., Kim H., Joo H.J., Jeong S.H., Park E.-C., Jang S.-I. (2021). Impact of changes in housing tenure and affordability status on depressive symptoms: evidence from a longitudinal study. J. Affect. Disord..

[bb0110] Leiner D.J. (2019). Too fast, too straight, too weird: non-reactive indicators for meaningless data in internet surveys. Survey Res. Methods.

[bb0115] Mason K.E., Baker E., Blakely T., Bentley R.J. (2013). Housing affordability and mental health: does the relationship differ for renters and home purchasers?. Soc. Sci. Med..

[bb0120] Ministry of Internal Affairs and Communications (2020). Population Census. https://www.e-stat.go.jp/en/stat-search/files?page=1&layout=datalist&toukei=00200521&tstat=000001136464&cycle=0&tclass1=000001136467&tclass2val=0.

[bb0125] Ministry of Internal Affairs and Communications (2021). Population Estimates. https://www.e-stat.go.jp/en/stat-search/files?page=1&layout=datalist&toukei=00200524&tstat=000000090001&cycle=7&year=20210&month=0&tclass1=000001011679&tclass2val=0.

[bb0130] Ministry of Land, Infrastructure, Transport and Tourism (2023). Housing Market Survey (in Japanese). https://www.e-stat.go.jp/stat-search/files?page=1&layout=datalist&toukei=00600630&tstat=000001017729&cycle=8&year=20231&month=0&result_back=1&tclass1val=0.

[bb0135] Ogawa K., Shimatani K., Iwayama R., Suzuki N. (2025). Association between higher mortgage payment-to-income ratio and greater psychological distress among high-income homeowners in Japan: a cross-sectional study. Prev. Med. Rep..

[bb0140] Ogawa K., Shimatani K., Endo K., Suzuki N. (2026). Associations between individual housing cost burden and depressive symptoms by relative income: a 13-wave panel study. Public Health.

[bb0145] Park G.-R., Jung Y. (2019). Housing insecurity and health among people in South Korea: focusing on tenure and affordability. Public Health.

[bb0150] Park G.-R., Seo B.K. (2022). Housing cost burden and material hardship among older adults: how do they influence psychological health?. Int. J. Geriatr. Psychiatry.

[bb0155] Park G.-R., Seo B.K. (2023). Multidimensional housing insecurity and psychological health: how do gender and initial psychological health differentiate the association?. Public Health.

[bb0160] Sakurai K., Nishi A., Kondo K., Yanagida K., Kawakami N. (2011). Screening performance of K6/K10 and other screening instruments for mood and anxiety disorders in Japan. Psychiatry Clin. Neurosci..

[bb0165] Singh A., Aitken Z., Baker E., Bentley R. (2020). Do financial hardship and social support mediate the effect of unaffordable housing on mental health?. Soc. Psychiatry Psychiatr. Epidemiol..

[bb0170] Wang Y., Mao Z., Wang D. (2023). Housing affordability and mental health in urban China: a cross-sectional study. Hous. Stud..

[bb0175] Zou G. (2004). A modified Poisson regression approach to prospective studies with binary data. Am. J. Epidemiol..

