# Supplementary material for: Housing affordability and psychological distress: Household measures mirror individual estimates but miss one in three at-risk adults
Source: Prev Med Rep. 2026 Jul 2;68:103556. doi: 10.1016/j.pmedr.2026.103556 (PMC13382060; doi:10.1016/j.pmedr.2026.103556)
Supplement: Supplementary file 1 — Supplementary material [file mmc1.docx]

**Supplementary Table 1. Prevalence ratios of psychological distress among adults by housing tenure and housing affordability using equivalized household income as the denominator for the household measure: a web-based survey in Japan, 2025**

|  | **Household housing affordability (equivalized)** | | **Individual housing affordability** | |
| --- | --- | --- | --- | --- |
|  | Unadjusted | Adjusted | Unadjusted | Adjusted |
|  | PR [95% CI] | PR [95% CI] | PR [95% CI] | PR [95% CI] |
| **Overall** |  |  |  |  |
| Affordable (< 30%) | 1.00 | 1.00 | 1.00 | 1.00 |
| Unaffordable (≥ 30%) | 1.35 [1.27, 1.45] | 1.13 [1.05, 1.21] | 1.31 [1.19, 1.43] | 1.17 [1.06, 1.28] |
| **Homeowner** |  |  |  |  |
| Affordable (< 30%) | 1.00 | 1.00 | 1.00 | 1.00 |
| Unaffordable (≥ 30%) | 1.54 [1.41, 1.69] | 1.19 [1.08, 1.32] | 1.53 [1.33, 1.76] | 1.33 [1.14, 1.54] |
| **Renter** |  |  |  |  |
| Affordable (< 30%) | 1.00 | 1.00 | 1.00 | 1.00 |
| Unaffordable (≥ 30%) | 1.04 [0.95, 1.15] | 0.99 [0.89, 1.11] | 1.02 [0.90, 1.14] | 1.01 [0.89, 1.14] |

Abbreviations: PR, prevalence ratio; CI, confidence interval.

Data from modified log Poisson regression with affordable housing (< 30% of income) as reference.

Adjusted models controlled for age, gender, marital status, educational attainment, employment status, household size, annual household income, population size.

Estimates were pooled from 30 imputed datasets using Rubin’s rules.

**Supplementary Table 2. Agreement between individual and household housing affordability measures among adults using equivalized household income as the denominator for the household measure: a web-based survey in Japan, 2025**

|  | **Individual housing affordability** | |
| --- | --- | --- |
| **Household housing affordability (equivalized)** | <30% | ≥30% |
| <30% | 5482.9 (89.1%) | 72.3 (15.6%) |
| ≥30% | 671.4 (10.9%) | 392.4 (84.4%) |
| Total | 6154.3 (100.0%) | 464.7 (100.0%) |

Values represent the pooled average counts and column percentages across the multiply imputed datasets.
